# Nanostructural changes in dentine caused by endodontic irrigants

**DOI:** 10.4317/medoral.18713

**Published:** 2013-03-25

**Authors:** Marta Barón, Carmen Llena, Leopoldo Forner, María Palomares, Cristina González-García, Manuel Salmerón-Sánchez

**Affiliations:** 1 DDS. Postgraduate program in Endodontics. Department of Stomatology. Universitat de València, (Spain); 2 MD, DDS, PhD. Professor. Department of Stomatology. Universitat de València, (Spain); 3 DDS. Associate Professor. Department of Stomatology. Universitat de València, (Spain); 4 MSc. Center for Biomaterials and Tissue Engineering. Polytechnic University of Valencia, (Spain); 5 PhD. Professor. Center for Biomaterials and Tissue Engineering. Polytechnic University of Valencia, (Spain)

## Abstract

Objective: To study nanostructural dentinal changes produced by endodontic irrigants. Study Design: Experimental study. Nanoindentations were performed on peritubular (PD) and intertubular dentine (ID) with an atomic force microscopy. Stiffness and adhesion force were determined before and after application of 5.25% sodium hypochlorite (NaOCl) and 17% ethylenediaminetetraacetic acid (EDTA). Normalized differences before and after treatment for stiffness and adhesion forces were calculated. A paired T-test was used to compare stiffnes and adhesion force before and after irrigants application. Results: After treatment with EDTA there was a 29.80% reduction in stiffness in ID and a 63.53% reduction in PD. Adhesion force was reduced by 21.22% and 8.21% respectively. After treatment with 5.25% NaOCI stiffness was reduced by 2.49% in ID and increased by 15.01% in PD. Adhesion force increased by 25.11% and 23.97% respectively. Conclusions: 17% EDTA reduced stiffness and adhesion force in ID and PD. Treatment with NaOCI at 5.25% had no significant effect on stiffness but did affect adhesion force in ID and PD.

** Key words:**Atomic force microscope, stiffness, adhesion force, peritubular dentine, intertubular dentine.

## Introduction

Knowledge of the mechanical properties of dentin is important for understanding how oclusal forces are distributed through the tooth and to predict how the absorption of these stresses is modified by age, pathological processes and restorative procedures (1-3)

The microstructure of the dentin-pulp complex consists of dentinary tubules which go from the pulp chamber to the dentino-enamel-junction in the crown portion and to the dentino-cementum-junction in the radicular aspect. And its microstructure consists of a hydrated type I collagen matrix reinforced with nanocrystalline carbonated apatite. The tubule lumens are about 1 µm in diameter and are surrounded by a 0.5-1.5 ?m hypermineralised layer of peritubular dentine which seems to be non-collagenous (2-6).

The atomic force microscope (AFM) is a mechanical-optical instrument that is able to detect forces at piconewtons level. It can scan the sample with a pyramidal tip, registering its topography, and produce indentations in dentin. The probe is connected to a microscopic flexible lever. When a nanoindentation is made, stiffnes of the studied material is registered, as well as the adhesion force, measured as the tip´s resistance to be extracted.

AFM can be used to specify the precise area for performing nanoindentations and to evaluate changes in stiffness and adhesion force in peritubular dentin (PD) and intertubular dentin (ID). ID is mainly responsible for the mechanical properties of dentin (1,3-8) because it is more widespread.

Irrigant solutions used in endodontic treatments can produce changes in biomechanical properties of dentin. Any change in the calcium/phosphate ratio alters the proportion of organic and inorganic components and consequently affects the characteristic hardness, permeability and solubility of dentin (9-14). Many studies have shown that different concentrations of chelating agents and citric acid can reduce dentin hardness (9) and this effect increases with exposure time (14). Although NaOCl is not a chelating agent, it can also modify the calcium/phosphate ratio in radicular dentin (14). NaOCI is a classic irrigating solution in endodontic treatment. Its main action is to eliminate organic residue in the smear layer and in the small and narrow canals that cannot be accessed by mechanical instrumentation. Other irrigating solutions with chelating action have been examined, such as EDTA, EDTA plus Cetavlon (EDTAC) and ethylene glycol-bis(beta-aminoethyl ether) (EGTA), among others (15-17).

As, in endodontic practice, many authors recommend the combined use of EDTA and NaOCI in the irrigation process, more knowledge about related changes that could interest dentin biomechanical properties is needed. The null hypothesis is that there are not biomechanical dentin changes in both ID or PD after treatment with EDTA or NaOCl at a nanostructural level. The aim of our study was to assess changes in stiffness and adhesion force in ID and PD caused by 5.25% NaOCI and 17% EDTA, using an atomic force microscope.

## Material and Methods

Human mandibular premolars (n=20) extracted for orthodontic reasons, were randomly selected and stored for a maximum period of 20 days in Hank´s Balanced Salt Solution at 4ºC. Cleaned teeth were introduced into epoxy resin (Buehler, Irvine, USA). A 1-2 mm thick disc of root dentine (3 mm below the most apical portion of the dentin-enamel junction on the buccal surface) was cut using an IsoMet Linear Precision Saw (Buehler, Irvine, USA) with a 15LC blade (Buehler, Irvine, USA) on a 0.381 thickness setting at 3000 rpm. Each slice were meticulously polished with P600, P800 and P1200 discs used consecutively (Microcut Silicon Carbide Grinding Paper, Buehler, Irvine, USA).

Samples were randomly distributed into two groups for the different treatments (n=10). The first group was submerged in 5.25% NaOCI for one minute. The second group was submerged in 17% EDTA also for one minute.

Nanoindentations were performed with a NanoScope Illa version 5.30r2 atomic force microscope (AFM) (Microscopic Digital Instruments, Santa Barbara, CA, USA) using a square pyramidal tip OTR8 (Veeco, Plainview, NY, USA) with a 0.57 N/m spring constant and a tip half angle of 35º on the buccal side. Calibration of the tip sensitivity was performed in the same conditions as the experiments with a flat surface sample made of the same material as the tip and was calculated to be 5.721 nm/V after at least 10 repetitions. Sensitivity of the tip was used to correct the deflection of the tip caused by the vertical movement of the sample without penetration. Stiffness and adhesion force were calculated with the AFM as previously described by Forner, et al. (18).

These experiments allows to get information about the local mechanical properties of the system, tests were performed in each sample (before and after treatment) in 200 points located on a 10x10 matrix, with rows and columns separated 10 nm from each other. Thus, stiffness is measured over a square of 1x1 µm2. The nanoindentation area was selected after several imaging steps for each specimen. First, a region with homogeneously distributed dentin tubules (free of defect or impurities) was selected using an optical microscope (400x) coupled to the AFM. Afterwards, several images of increasing resolution were acquired (AFM) until a single dentin tubule in a 5x5 µm area was visualized. The nanoindentation areas (intertubular and peritubular dentin) were then selected from this high-resolution image. Nanoindentation experiments provide the adhesion force and the stiffness for each sample as the mean value of 200 points (100 in both ID and PD). These measurements can be used to assess adhesion force and stiffness.

Adhesion force and stiffness measurements of the 200 nanoindentations were calculated for each sample before and after treatment. The normalized difference between the two magnitudes (before and after treatment) was calculated. Mean values before and after treatment was performed by paired T-test. Statistical significance was assessed at p < 0.05.

## Results

**Figure 1:**
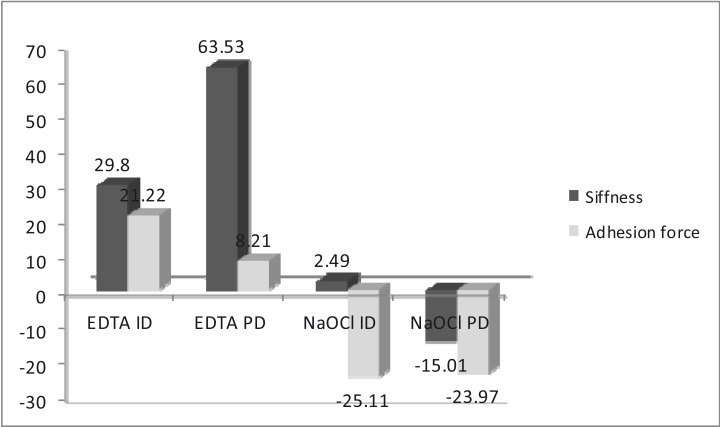
Normalized differences (percentage) for stiffness and adhesion force before and after treatment on peritubular (PD) and intertubular (ID) dentin. EDTA: ethylenediamine.tetraacetic acid. NaOCl:, sodium hypoclorite.

The mean values of normalized differences for stiffness and adhesion force for both ID and PD were represented in figure 1. The samples treated with 17% EDTA showed significant reduction in stiffness (p<0.05), which was higher in PD (63.53%) than in ID (29.80%). Adhesion force was also significantly reduced in both ID and PD areas after treatment (p<0.05) ( Table 1).

**Table 1:**
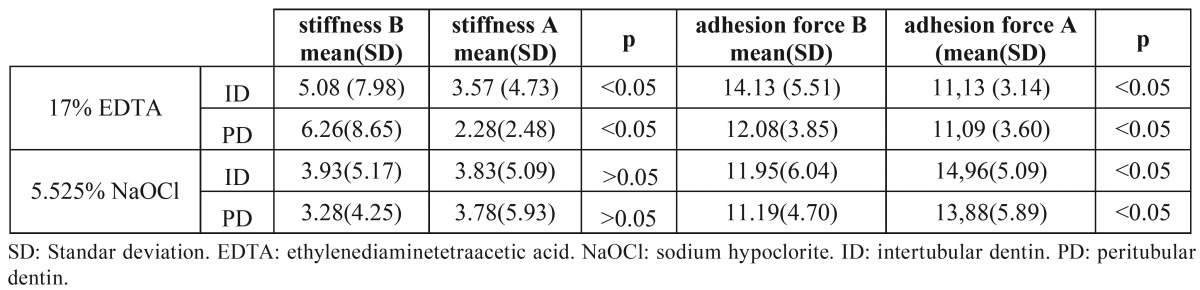
Mean and SD of stiffness and adhesion force before (B) and after (A) treatment. Statistical signification: p<0.05.

The samples treated with 5.25% NaOCI showed no significant differences in stiffness before and after treatment in ID or PD (p>0.05) whereas adhesion force increased 25.11% in ID and 23.97 in PD (p<0.05) ( Table 1).

## Discussion

In our study we used samples from buccal root dentin from mandibular premolars in order to standardize the samples since, according to results obtained by Brauer, et al. (19), buccal coronal dentine have consistently lower elastic modulus and hardness than lingual, and in the root dentine the lingual side is less hard than buccal (opposite to crown dentine) (19).

There is no information available in the current scientific literature about changes in stiffness and adhesion force at nanostructural levels in intertubular and peritubular dentin after the use of different products to irrigate root canals during biomechanical preparation. The use of AFM is a suitable technique for this study and has been used previously to evaluate the effects of other products on dentin (20,21), as it enables the changes produced in PD and ID (which have different structures and compositions) to be explored independently (19). It also provides information on the changes caused on the same area in the sample before and after treatment.

AFM measures the mark left by the indenter tip (coupled to the microscope) where it comes into contact with the dentin (entry stiffness), providing information on the capacity of the dentin to withstand the force applied by the nanoindentor, that is, dentin stiffness. The resistance offered by the tip as it comes off the dentin provides the value known as adhesion force (19).

In the conditions of our experiment, the null hypothesis was rejected because 1 minute application of 17% of EDTA reduces stiffness and adhesion force in both ID and PD dentin, as well as 1 minute application of 5,25% NaOCl increases adhesion force.

The reduction in stiffness and adhesion force found after applying 17% EDTA for 1 minute may be due to demineralisation. This reduction is more significant in PD because PD is more mineralised than ID. Other techniques used to evaluate the effect of EDTA at the same concentration on dentin are microindentation techniques which observe reductions in dentin hardness (9,10,13-17). Several studies have analysed the microhardness of dentin with various agents and it has been reported that 10% citric acid and 15% EDTA significantly reduce dentin microhardness (15) . It has also been shown that 17% EDTA not only reduces microhardness in radicular dentin but also increases roughness (17). Another study confirms that 17% EDTA increases roughness but with no significant reduction in dentin wettability, favouring the adhesion of sealants used to fill the tubes (22). Other studies show that 17% EDTA influences dentin flexing stress (23).

NaOCI is essential for eliminating organic components during root canal treatment (24). 5.25% NaOCI decreases Young’s modulus of dentin and its flexing stress (14). The use of 2.5% NaOCI also reduces flexing stress but does not change the elasticity modulus which is attributed to an exposure time that is short enough not to alter the mechanical properties of dentin (23). Many other studies have evaluated the effect of NaOCI using microindentation techniques (9,13,25-27), finding a reduction in dentine elasticity after treatment. In our study, PD showed a 15.01% increase in stiffness which coincides with above mentioned studies for overall dentine. Similarly, greater stiffness was associated with increased adhesion force of the tip to the dentin. These results can be explained by the fact that NaOCI mainly dissolves the organic component in dentin, which is responsible for its elasticity thereby giving rise to increased stiffness and adhesion force.

We can conclude that both 17% EDTA and 5% NaOCI cause nanostructural changes in intertubular and peritubular dentin. Further studies on these lines are needed to establish the effects of other concentrations and other application times and to determine the biomechancial significance of these changes.
